# Perceptions of isolation during facility births in Haiti - a qualitative study

**DOI:** 10.1186/s12978-019-0843-1

**Published:** 2019-12-27

**Authors:** Alka Dev, Chelsey Kivland, Mikerlyne Faustin, Olivia Turnier, Tatiana Bell, Marie Denise Leger

**Affiliations:** 1 Geisel School of Medicine at Dartmouth College, Hanover, United States; 2 Dartmouth-Hitchcock Medical Center, 1 Medical Center Drive, 330W Borwell, Lebanon, NH 03756 United States; 3 Anthropology, Dartmouth College, Hanover, United States; 4 GHESKIO, Port-au-Prince, Haiti; 5 SUCO, Port-au-Prince, Haiti

**Keywords:** Focus groups, Birth experiences, Facility births, Haiti

## Abstract

**Background:**

Haiti’s maternal mortality, stillbirth, and neonatal mortality rates are the highest in Latin America and the Caribbean. Despite inherent risks, the majority of women still deliver at home without supervision from a skilled birth attendant. The purpose of this study was to elucidate factors driving this decision.

**Methods:**

We conducted six focus group discussions with women living in urban (*N* = 14) or rural (*N* = 17) areas and asked them questions pertaining to their reasons for delivering at a facility or at home, perceptions of staff at the health facility, experiences with or knowledge of facility or home deliveries, and prior pregnancy experiences (if relevant). We also included currently pregnant women to learn about their plans for delivery, if any.

**Results:**

All of the women interviewed acknowledged similar perceived benefits of a facility birth, which were a reduced risk of complications during pregnancy and access to emergency care. However, many women also reported unfavorable birthing experiences at facilities. We identified four key thematic concerns that underpinned women’s negative assessments of a facility birth: being left alone, feeling ignored, being subject to physical immobility, and lack of compassionate touch/care. Taken together, these concerns articulated an overarching sense of what we term “isolation,” which encompasses feelings of being isolated in the hospital during delivery.

**Conclusion:**

Although Haitian women recognized that a facility was a safer place for birthing than the home, an overarching stigma of patient neglect and isolation in facilities was a major determining factor in choosing to deliver at home. The Haitian maternal mortality rate is high and will not be lowered if women continue to feel that they will not receive comfort and compassionate touch/care at a facility compared to their experience of delivering with traditional birth attendants at home. Based on these results, we recommend that all secondary and tertiary facilities offering labor and delivery services develop patient support programs, where women are better supported from admission through the labor and delivery process, including but not limited to improvements in communication, privacy, companionship (if deemed safe), respectful care, attention to pain during vaginal exams, and choice of birth position.

## Plain English summary

Fewer women in poorer countries give birth in hospitals or health centers, despite the fact that giving birth in facilities with skilled birthing attendants is globally promoted as the standard of care in maternal health. In Haiti, for example, the majority of women deliver at home without the assistance of a trained provider; Haiti also has the highest rate of maternal mortality in Latin America and the Caribbean. The aim of this study was to understand the reasoning behind a Haitian woman’s choice to deliver at a facility versus at home and to compare and contrast women’s experiences in different birthing environments. We carried out six focus group discussions; three in a rural setting and three in an urban setting. Women were recruited for the focus groups with the assistance of local prenatal clinics in the selected communities. We found that while many women knew that a facility could support a safer birth with less complications, there were also a number of negative experiences of facility births; women described being isolated, neglected, physically restrained, and/or not receiving compassionate touch. Our study supports what others have found with regard to respectful maternity care, but we were particularly struck by the sense of isolation and lack of compassionate touch in comparison to Haitian home births, where women deliver with traditional birth attendants (locally known as *fanm chay*) and receive hands-on attention, care, and comfort. We found that most women who initially trusted facility staff to address their medical needs subsequently lost that trust when they experienced neglect, mistreatment, and isolation. Their experiences also impacted their future choices for delivery and the advice they gave to other pregnant women. Therefore, given these findings, we propose that facilities in Haiti need to focus on improving the quality of patient-provider interactions and to understand how to best foster a supportive environment during labor and delivery. Potential low-cost interventions include sensitivity and communication training for facility staff, appointment of patient navigators, and allowing the woman her choice of birth companion. Further research is needed to determine best practices for reducing maternal mortality in Haiti.

## Background

Labor and delivery are extremely high-risk periods for mothers and babies, worldwide. Every year, at least a quarter of the estimated 293,000 maternal deaths, 4 million neonatal deaths, and 2.6 million stillbirths occur during delivery [1, 2]. Skilled birth attendants—including doctors, nurses, and midwives—can reduce maternal and neonatal morbidity and mortality by safely handling deliveries, quickly recognizing complications, and referring women to emergency obstetric care, if needed [3]. Globally, the rate of having a skilled birth attendant present at birth has increased from 62% (2000–2005) to 80% (2012–2017) [4]; however, the rates of skilled birth attendance continue to be low in countries like Haiti, where nearly 60% of all women deliver at home, and the majority of these with the help of a traditional birth attendant, or *fanm chay* [5]. Skilled birth attendance in Haiti is only available in health-care facilities, including hospitals and health centers.

Thaddeus and Maine’s framework of the “three delays” has been applied globally to understand the major contributors to maternal death beyond medical causes, and includes: delay by the woman and/or her family in deciding to seek care (due to socio-cultural, socioeconomic, geographic, financial, and institutional factors); delay in reaching an adequate health-care facility (due to distance, transport, cost factors); and delay in receiving adequate care at that facility (due to staffing, infrastructure, and service-delivery-related factors) [6]. To address the limitations posed by these delays, different models are employed to encourage timely facility births. In Zambia and Malawi, maternity waiting homes (MWHs) have been expanded across rural areas so women in remote and hard-to-reach communities can stay near the facility shortly before their delivery date [7, 8]. Some health-care facilities in Haiti have also built MWHs to reduce delays in seeking and receiving skilled care during labor and delivery [9, 10]. MWHs intend to relieve the burden of finding transportation and allow for appropriate antepartum and intrapartum monitoring. Other approaches have focused on improving the quality of maternal care [11]. The World Health Organization (WHO) Safe Childbirth Checklist, for instance, was designed to improve the delivery of essential maternal and perinatal care practices and includes critical actions for preventing maternal and neonatal death and intrapartum stillbirths in health-care facilities [12].

The global movement to promote respectful maternity care and reduce instances of disrespect and abuse has also made an unequivocal case for the importance of respect, dignity, and privacy in the case of facility births [13]. A systematic review by Bohren and colleagues showed that mistreatment of women during childbirth—from physical and verbal abuse to stigma and discrimination—is common throughout the world [14]. The evidence regarding the mistreatment of women during labor and delivery in facilities continues to escalate in strikingly similar ways across the world [15–17]. While it is important to increase the proportion of women who choose to deliver with skilled birth attendants, we will need to go beyond just clinical interventions to improve women’s experiences of care during childbirth. Women who are mistreated during childbirth, or who know of other women who are mistreated, are less likely to choose a facility birth [18].

In Haiti, where the majority of women deliver at home, we wanted to elucidate the motivating factors driving women to choose to give birth at a health facility versus at home, and to document their past experiences delivering in a facility setting. We hoped that our findings would result in strategies that could help more Haitian women to choose a facility birth with a skilled provider.

## Methods

The study methodology is reported using the consolidated criteria for documenting qualitative research (COREQ) [19]. See Additional file 1.

### Design and participants

In March 2018, we conducted a qualitative study using focus group discussions (FGDs) with women in rural and urban settings in Haiti. A focus group is a qualitative research method in which one or two trained moderators lead a discussion with 6–8 participants to explore a research topic in a group setting [20]. Rather than one-on-one interviewing, FGDs rely on participant interactions to yield information that can capture shared and divergent experiences across the group with regard to the research question. We divided the study across rural and urban settings in order to elicit perspectives across a salient social divide in Haiti. We also wanted to know if reasons for delivering in a health-care facility and experiences of facility versus home births would vary across the rural/urban divide. Tables 1 and 2 present the participants’ parity and place of birth (recent or current pregnancy), organized by rural and urban settings. No one refused to participate or dropped out in either setting. Women in the urban setting tended to have lower parity and were younger. Rural women had more experience with birth and had more experiences to offer during the FGDs. We use the terms health facility and hospital interchangeably as the latter was used by women most frequently, even when referring to a health center.

**Table 1:** Parity among Participants

	Parity	Rural	Urban
	0 (1st Pregnancy)	0 (0%)	5 (35.7%)
	1	7 (41.2%)	3 (21.4%)
	2	5 (29.4%)	4 (28.6%)
	3	1 (5.9%)	1 (7.1%)
	4+	4 (23.5%)	1 (7.1%)
TOTAL		17	14

**Table 2:** Place of Birth for Most Recent Pregnancy among Participants

		Rural	Urban
	No prior live birth	0 (0%)	7 (50%)
	Home only	8 (47.1%)	4 (28.6%)
	Hospital only	8 (47.1%)	2 (14.3%)
	Home and Hospital	1 (5.9%)	1 (7.1%)
TOTAL		17	14

We conducted a broad study exploring a wide array of potential reasons for delivering at a facility or at home (e.g., cost, transportation, crime/insecurity, religious and cultural values regarding birth, history of prenatal care, and other individuals involved in decision-making) and the range of women’s experiences with facility and home births. In accordance with our study design, we purposively sampled expectant or postpartum women in three rural communes of southern Haiti and in three downtown districts of Port-au-Prince. In the rural setting, we had recently completed a census of households in the three communes that were being served by a local health center [21]; these data were used to identify women who were either currently pregnant or who had delivered in the previous year, and the site of their delivery. Working with staff from the health center, we invited 17 rural women to participate in three FGDs. They were grouped according to the place of delivery of their most recent birth: six women who had delivered at a health-care facility (Rural #1–6), five women who had delivered at home (Rural #7–12), and five women who were pregnant at the time of the study (Rural #13–17). Two of the pregnant women had previously given birth at a health-care facility; three had previously given birth at home.

In Port-au-Prince, we worked with GHESKIO, a local public health organization that provides antenatal care in three urban slums. As part of a prior Zika study, the group had enrolled 1500 pregnant teens and women into antenatal care, many of whom had since delivered [22]. GHESKIO staff suggested that we target our FGDs by geographic area, rather than place of birth, to make it easier to recruit participants. We therefore invited 14 women to take part in three FGDs, grouped according to their place of residence (Urban #1–14). All three groups had a mix of pregnant women and women who had recently delivered. In addition, the groups included some who had given birth at a health facility, some who had given birth at home, and others who had had some births at a facility and some at home. Half of the women had given birth recently and the other half were anticipating their first live birth. Although primiparous women had no prior live birth experiences, we explored the perceptions they held about a home versus facility birth and how those perceptions factored into their decision regarding the place of their upcoming birth.

### Data collection

Based on a review of published qualitative research on maternal health-seeking behavior in Haiti and our own academic training and professional experiences, we developed our question guide and study procedures (see Additional file 2: Question Guide for Focus Groups) [23–25]. The FGDs were held in a private room at our collaborating organizations’ clinics and were conducted in Haitian Creole, recorded, and lasted between sixty and ninety minutes. All focus groups were led by CK, a professor of anthropology, who is fluent in Haitian Creole and, having lived in Haiti for three years and conducted research there for the past decade, accustomed to the sociocultural mores of the country. The participants were informed of the study aims and procedures, including audio recording, and their verbal consent was obtained prior to initiating the group discussions. We opted for oral consent in order to communicate with all participants regardless of literacy levels (see Additional file 3: Information Sheet/Consent Form). We also had written forms available for participants who might request them, but we did not have any requests. During the FDGs, the participants were identified by number rather than name, and they were informed that they could discontinue the recording at any point or refuse to answer any questions for any reason. In the rural setting, ML, a community representative, assisted CK in the FGDs; in the urban setting, OT and MF, two staff psychologists at GHESKIO, assisted CK. Both OT and MF work with the GHESKIO antenatal care clinic and provide mental health support to women who are HIV positive. They are both Haitian, native speakers of Haitian Creole, and were available to clarify questions for participants and to accompany participants if they wished to suspend their participation. All researchers had received training in qualitative research methods and ethics. All of the researchers and participants were women. There were no prior relationships between the researchers and participants. During the discussions, CK and ML also shared their pregnancy experiences as a way to establish trust with the group; no other personal information was shared by the researchers. Aside from the researchers and participants, no one else participated in the FGDs or was present in the room. No repeat meetings or interviews were conducted.

The discussions commenced with introductions, where the facilitators identified themselves, welcomed the women to the group, reviewed the procedures and aims of the study, and elicited oral consent. In line with cultural norms, we asked the women how they were doing, how their families were doing, and what their activities were. The women then offered details about their recent birth or expectations for their pending labor, including number of pregnancies, place of delivery, and overall assessment of their prior experiences. We then asked the overarching research question, “Where do you prefer to give birth, and why?” The interview continued with secondary questions to elicit a deeper and broader picture of their reasons for preferring facility or home births, their experiences and perceptions of facility or home births, and their evaluations of the facility or home birth. As this was a qualitative study, we allowed the conversation to develop in an open-ended way and took cues from the participants about which topics and questions to explore. This resulted in a broad discussion regarding the perceptions and evaluations of home and facility births in Haiti; however, for the purposes of this paper, we focused our analysis to a specific contradiction that readily became apparent in all of our groups, which was that women simultaneously expressed a preference for a facility birth while also relaying negative prior experiences in facilities. We explored this contradiction further through sub-questions that elicited more details about how the facility birth experience differed from the home birth experience, as well as what women liked and disliked about the birthing experience in each setting. We then asked the women how they would address the limitations or negative aspects of the health facility birthing experience. The FGDs ended once the researchers had a full understanding of birthplace experiences and no new information was being provided to inform the aforementioned contradiction. The full interview guide is shown in Additional file 2.

### Data analysis

The recorded discussions were first transcribed into Haitian Creole, then translated into English. The transcriptions and translations were not returned to participants, but rather verified by CK. Data analysis of the interviews was carried out according to grounded theory methods [26]. Upon transcription and translation, we reviewed the FGD texts and developed a coding scheme based on prominent and relevant concepts (Table 3). Each author took responsibility for coding different sets of data using QDAMiner Lite, an open-source software package for qualitative analysis [27]. AD took the primary responsibility for coding discussions from the rural site and CK coded discussions from the urban site. Similar codes were grouped together in sub-categories and domains. CK and AD discussed codes to determine broad relationships between choice of place of prior births or, for pregnant women, where they intended to give birth and why. All authors reviewed and discussed relevant coded text and clarified translation issues. Participants were not contacted to provide feedback on the findings due to limitations posed by literacy and challenges in re-locating participants, especially in the urban setting. We recognize this as a limitation of the study.

**Table 3:** Coding Scheme

**Domain: Quality of Care - hospital birth** Perceptions of proper/normative birth Perceptions of good doctor/hospital Experiences at hospital (for birth and other) **Domain: Quality of care- home birth** Perceptions of good/bad midwife (*fanm chay)* Experiences with midwife (*fanm chay*) **Domain: Reasons for hospital birth** Complications History of hospital care Precipitous labor Location Planning Cost Referral (by a doctor or clinic) Family Advice	

## Results

All of the women we interviewed acknowledged that there are significant benefits to a facility birth, including the capacity to address complications and emergencyt care needs. The women were concerned about the potential for having a difficult labor or losing a baby based on their own or other family members’ and friends’ past experiences with pregnancy complications. During our discussions, several women reported pregnancy losses, stillbirths, and infant deaths. In the rural setting, one participant reported a late-term miscarriage; one woman reported a stillbirth at a public hospital; one woman reported an infant death after a home birth; and one woman who gave birth to seven children at home reported a stillbirth and three infant deaths. In the urban setting, two of the women expecting their first child reported having previously had a late-term miscarriage; one reported a stillbirth at home; one woman had a stillbirth at the hospital; and another woman suffered a neonatal death after giving birth at home.

The most common reasons given by women who decided to give birth at a hospital or health center were the development of a pregnancy complication and the reduced risk for a pregnancy loss, illness, or death. Potential complications were usually identified by the woman herself, and then confirmed by a provider who advised her to follow-up and deliver at a facility. There was consistency in reasons to seek a facility birth, regardless of an urban or rural status.

*“When I was pregnant, I chose to go to give birth at the hospital because my due date was changed. The delivery period didn't arrive yet, but I had some liquid flowing out of me. They sent me to a private clinic, the doctor did an ultrasound and told me to go to the hospital directly.”*

- (Rural #1, postpartum woman with hospital-birth experience)

*“The reason I chose to give birth at the hospital was because when I was pregnant, I was sick, my feet were swollen, I became anemic. Every time I went to the [clinic], the doctor told me not to stay at home when I would have pain; always go to the hospital because if it becomes dangerous for me, I can find a doctor to help me. That's why I gave birth in hospital.”*

- (Rural #4, postpartum woman with hospital-birth experience)

*“I followed here in the [clinic] and every month the doctor gave me a vaginal exam, but he told me that I should go to the hospital because my whole body was swollen. He also told me that I had eaten too much salt, so I went to the hospital.”*

- (Rural #3, postpartum woman with hospital-birth experience)

*“Yes, because I'm sickly, I would not have risked giving birth at home with a* matwon [another word for famn chay]*; I would always go to the hospital so that the doctor could see what is necessary for me, because as soon as he sees what I need, he will give it to me.*”

- (Rural #2, postpartum woman with hospital-birth experience)

Two women, who had recently given birth at home, expressed that a facility would have been preferred if there had been complications.

*“My dream is to go to the hospital because childbirth can be dangerous when you stay home. If the baby comes normally, you can give birth at home or even the roadside [...] But if the baby does not come out normally, you have to go to the hospital because sometimes there can be a caesarean section and other problems.”*

- (Urban #3, postpartum woman with home-birth experience)

*“If I have difficulties at home, I will go to the hospital, but otherwise I will stay at home. However, if during labor, I have contractions early in the morning and after 8 hours of labor the baby does not arrive yet, I will immediately go to the hospital because I'm not used to staying too long in labor or having severe pain that I cannot bear.”*

- (Rural #8, postpartum with home-birth experience)

Among pregnant women, the risk of developing a complication was also an adequate reason to decide to give birth at a facility.

*“The* fanm chay *does not have the means to see the child inside your belly. The doctor has a device to see if the baby is leaning to the right or left.”*

- (Urban #5, pregnant woman with prior hospital-birth experience)

*“I plan to give birth at the hospital because, from time to time, I feel abdominal pain, so it would be better if I had my child in the hospital.”*

- (Rural #16, pregnant woman with home-birth experience)

*“I think that hospitals have more benefits, because when you are coming to give birth, they look if there is a problem with the baby, and they look with a medical examination. They see what the problem with the baby is and they fix it… the hospital is better for you because there are several doctors and they can transfer you to another hospital. That's why I encourage you to go to the hospital, ladies and me, who used to give birth at home. I have always liked to stay at home, but I do not keep this habit anymore because I realize that things have changed.”*

- (Urban #12, pregnant woman with home-birth experience)

*“Nowadays, you sometimes see a woman giving birth to a child with a malformation, a big head. But if you had gone to the hospital, we would have seen in time how the child is. There would have been some prevention. That's what interests me and pushes me to come to the hospital.”*

- (Urban #3, pregnant woman with hospital-birth experience)

In this last example, the woman is specifically referring to cranial malformations due to the Zika virus, which was then a concern in Haiti. She is expressing the importance of visiting the hospital for prenatal care, as well as for delivery of the baby, in order to detect abnormalities that would result in complications during labor. The understanding that the hospital setting provided the experts and tools to detect and manage problematic conditions during pregnancy and labor proved to be a powerful motivator for desiring facility-based care.

We also found that, despite a shared desire for a facility birth, most women simultaneously expressed an unfavorable perception of the facility birthing experience. We identified four key, thematic concerns that underpinned women’s negative assessments of the facility birth: being left alone, feeling ignored, being subject to physical immobility, and experiencing a lack of compassionate touch/care. Taken together, these concerns articulated an overarching sense of what we call “isolation” -- that is the feeling of being isolated in the hospital and being isolated during delivery. The experience of isolation diminished a woman’s desires for a hospital birth and was often contrasted to the care received from a *fanm chay* during a home birth.

Exemplar narratives that elaborate upon the thematic elements of isolation are provided below, followed by an articulation of the consequences these elements had on a woman’s choice of birthplace.

### Theme 1: being left alone

Women frequently noted being left completely alone during labor in the delivery room and usually at the time of the delivery, which created a sense of isolation. Some women were particularly critical of being denied access to caretakers and the lack of any care during and after delivery.

*“In the hospital, you are alone, you are put on the delivery table, you are alone, you have no one to keep you company.”*

- (Urban #7, pregnant woman with hospital-birth experience)
  *“Being alone on the delivery bed at the hospital is the worst experience. When you are in the big room with contractions, your parents stay with you, but when you are going to the delivery room, you are alone.”*
- (Rural #3, postpartum woman with hospital-birth experience)
  *“You are alone on the delivery bed; they will not let someone help you. When I was on the delivery bed, I sent someone to call the doctor to tell him that the baby's head is coming out, because it was midnight and they had fallen asleep in their chairs. I told the nurse that the baby was coming out and she woke up bewildered and told me to stop pushing and took the baby.*”
- (Rural #4, postpartum woman with hospital-birth experience)

*“When I took a look, I saw the doctor sleeping. They don’t even let you see the baby right away.”*

- (Rural #5, postpartum woman with hospital-birth experience)

*“They always took good care of me when I gave birth at the hospital. After giving birth, they bathe the baby and bring him to me... But now there are some hospitals that are problematic. Sometimes when you go to give birth, you are left in the courtyard screaming, and there is no one to pay attention to you. […] Not only at the general hospital, in all hospitals. I do not know if it is a lack of personnel, if it is the negligence of the doctors, but pregnant women suffer a lot.”*

- (Urban #9, postpartum woman with hospital-birth experience)

### Theme 2: feeling ignored

In addition to being left alone, women delivering at a health facility felt that being ignored was further manifested by feelings of dismissal and the denial of specific needs or requests. Women recounted experiences that showed that even when they were able to speak with a staff person, they were unable to receive appropriate care or were not given reasons for being denied their request.

*“When I could not continue, I told them that I needed to rest, I was told no, that I have to walk and walk again...They will not let your parents in. My mother wanted to get in. I bled a lot. My mother asked the nurse to let her in to help me, but the nurse said no.”*

- (Rural #5, postpartum woman with hospital-birth experience)

*“You cannot find even some water if you need it, because the doctors are not doing that for you. They just sit and wait for the baby's head to come out to ask you to stop pushing to take the baby.”*

- (Rural #1, postpartum woman with hospital-birth experience)

*“*…*when the shoulders appeared, I supported her head. I screamed and I called the nurse, she was in a chair and she came to get the baby.”*

- (Rural #2, postpartum woman with hospital-birth experience)

*“I went to the hospital, and they sent me home. When I arrived at home, I still had flowing liquid. So, I went back to the hospital, they took me to the emergency room, and told me either I undergo a caesarean section or I may die in this case. I was obliged to pray to God. I finally gave birth.”*

- (Rural #1, postpartum woman with hospital-birth experience)

### Theme 3: being subjected to physical immobility

Women also experienced isolation by being subjected to physical immobility. They disapproved of the restrictions placed on movement in a facility and, at times, the experience of being tied down during birth. Not being able to move and giving birth on a delivery table, or being tied to the table, were experiences that were seen as being unique to the facility setting.

*“They lie you down with both of your legs up and immobilized. So even if you have pain, you are not able to move a lot.”*

- (Rural #1, postpartum woman with hospital-birth experience)

*“I lost all my strength because of the way I was placed. I was put in the back position. I could not find any place to put my feet, to be able to resume force*.*”*

- (Rural #4, postpartum woman with hospital-birth experience)

*“I was moving too much, so they tied my legs down to immobilize them. In order to sit up, they had to pull me by both arms. I didn't have strength anymore to push the baby.”*

- (Rural #5, postpartum woman with hospital-birth experience)

*“They put you in a position so that the doctor can see your vagina, in order that the doctor may be comfortable… And when you have contractions, it would be better that someone held you. When they tie you down, it is traumatic.”*

- (Rural #4, postpartum woman with hospital-birth experience)

One woman related a harrowing story of being bound to the delivery table and having to break the bindings to deliver her own baby, although she did acknowledge that the attending doctor was apologetic afterward:

*“I broke the hand restraint. I told myself that I have to give birth to this child. I could not let him die. Then the doctor arrived, and he took the baby, and he also apologized to me because he told me he was very tired... I had to pull with all my strength to [release my arms and] grab the baby. And he [the doctor] knelt before me to apologize… There are two iron bars and a space between to put your feet. They tied my hands and my feet to the bars. When I had the feeling that the baby was coming, I do not know if it was God who put this strength in me, but I ripped off the hand restraint with great force, and I sat up to push.”*

- (Rural #2, postpartum woman with hospital-birth experience)

### Theme 4: lack of compassionate touch

Another reason women gave for not choosing a facility birth was the lack of compassionate touch from hospital staff, particularly in comparison to the hands-on support of a *fanm chay* who oversees home births. Many women valued the way in which the *fanm chay* physically held and supported them as they labored.

*“During those moments you are alone. You have no one to support you, whereas with the* fanm chay, *you may have someone to hold you, to support you. But at the hospital you don't have that kind of support.”*

- (Rural #4, postpartum woman with hospital-birth experience)

*“When you're with a* fanm chay*, there's someone who is holding your knees, helping you sit steady on the stool so you can find the force to push.”*

- (Rural #7, postpartum woman with home-birth experience)

In two FGDs, the expression, “hold you, so you can push,” was offered and met with much agreement among the women present. In addition, many women spoke of the massages and caresses given by midwives as a crucial act that eased pain and increased comfort, as well as provided emotional support.

*“Yes [I like the home birth] because you see the* fanm chay *and she gives you a massage on your belly and it makes you feel good.”*

- (Rural #9, postpartum woman with hospital- and home-birth experiences)

*“Well, when the* famn chay *arrived, she looked at me and she touched my belly, and she told me that I had contractions and could push. She massaged my belly when the pains came time and again, and I would feel a little better, and then I gave birth.”*

- (Rural #11, postpartum woman with home-birth experience)

*“*Fanm chay *are good. I liked the way she treated me. I was still in a lot of pain, but after the massage, the placenta came out from my vagina and the pain stopped.”*

- (Rural #10, postpartum woman with home-birth experiences)

The lack of physical and supportive attention to the laboring mother was often noted as missing in a facility birth. The desire for this compassionate touch in a facility was underscored by the women’s painful experiences with physical contact during medical procedures—most notably, the vaginal exam.

*“When you go to the hospital, the pain is worse, the pain.”* [CK asks, *“Why?”*]

*“Because you get vaginal touch, and that’s the only way [the staff’s] hands touch you.”*

- (Urban #4, pregnant woman with hospital- and home-birth experiences)

*“In the hospital, you have the vaginal exam to see how many centimeters you’re at and then you are told to go for a walk. You will not find anyone to touch and support you if you need it.”*

- (Rural #3, postpartum woman with hospital-birth experience)

### Consequences of isolation

We found that negative facility-birth experiences affected a woman’s decision as to where to have her birth in the future and weighed against the benefit of potential lifesaving interventions; however, many women continued to express a desire for a facility birth, although they wished the experience could be ameliorated to address the sense of isolation. Positive interventions suggested by women included: support from other people, being able to talk to a provider, having the father present, being given sound advice, and having a bed throughout labor.

*“The experience I had at the hospital that led me to choose the* fanm chay *was that I was left on the gynecological table, it was up to me to get by. I found no support from anyone.”*

- (Rural #4, postpartum woman with hospital-birth experience)

*“I had my first child in the general hospital, and I was well received, but now the situation has changed. There are state hospitals that do not give good service because of the [lack of] money. I had my second child in the [French] hospital and the doctor was gone. I had my child alone in the emergency room despite having paid 5000 gourdes* [~$78]*. Even after my delivery, we could not reach the doctor on the phone. So, I concluded that all hospitals were doing bad service…I almost lost my child. Things have changed in hospitals.”*

- (Urban #14, pregnant woman with hospital-birth experience)

*“When I feel that I am really not well, I go to the hospital to take medication, but for a pregnancy, I do not go there. When I give birth, I need the father of the child there, that’s all I need.”*

- (Rural #13, pregnant woman with home-birth experience)

*“Doctors come late in the day and despite this only a few people are consulted and at a certain time, they just close and leave, and the others that stay do not do anything, and they do not help you. And, so for that, I do not go there anymore.”*

- (Rural #9, postpartum woman with hospital- and home-birth experiences)

*“I have a sister who was giving birth in the hospital, when she arrived, she was put on the gynecological table...the cervix was not yet dilated. She was asked to walk and suddenly she gave birth on the ground. She lost strength and fainted. She was given an infusion. At the same time, if it was at home, the time to call a doctor or bring her to the hospital would have made things difficult.”*

- (Rural #17, pregnant woman with home-birth experience)

*“What we need to change in the hospital is for them to welcome the laboring woman. They should give them a bed because it is possible that the person cannot continue to walk all by herself. They should give the woman a bed during the whole day she is laboring…. At the hospital, it’s only when you are about to deliver that you are given a bed.”*

- (Rural #2, woman with home-birth experience)

## Discussion

During the FGDs, we learned that women had many different types of birthing experiences. There were: women who had delivered both at home and in a facility for different pregnancies; women who had exclusively delivered at home or in a facility for all their births; women who had wanted to deliver at a hospital but never made it; and women who had wanted to deliver at home, but had to be taken to a hospital due to a complication. There were also pregnant women who had given birth previously and who wanted to choose their place of birth based on an earlier experience; others who were pregnant for the first time and clear about their place of delivery; and those who did not know yet. In short, we realized that women did not fall into one, single category in terms of where they chose to give birth, or even in terms of the factors leading them to a given decision. We found this to be somewhat surprising, as other studies on maternal health categorized women into groups based on their discrete choices for place of delivery, and presumably the choices were consistent across all pregnancies [28–30]. Instead, we found that the same woman may make different choices for different pregnancies, depending on their circumstances and context.

What was most consistent in our study was that the hospital or health center was the preferred option, particularly in cases of suspected and confirmed complications. In those latter instances, women would often seek medical care in response to a complication and then be advised by a provider to deliver at a facility. There was enough trust in and access to a provider to seek care for complications. Further, the women who went to a facility also perceived that the facility would be able to manage their complication(s). Similarly, one study in southern Haiti concluded that prenatal care was associated with the ability of a woman to recognize pregnancy complications, and that women who better perceived symptoms were more likely to seek care [31]. However, the participants in our study also expressed a negative perception of what type of care they would receive in a facility. The anecdotal report from one woman about being physically immobilized was extreme, but it fit within the premise that there is an overall lack of attention and compassionate care for birthing women in a facility. Thus, women appeared to be consistently weighing the pros and cons of giving birth in a facility, e.g., a safer birth but in a colder and less compassionate environment, versus at home, surrounded by loved ones and the dedicated attention of a *fanm chay*. Women’s autonomy in decision-making to seek care has been associated with higher hospital birth rates, but it is possible that negative experiences dampen this association if women perceive the choice to be a poor one [32].

Although we found that the rural/urban division was relevant in some respects (e.g., costs and challenges of transportation and concerns about crime and insecurity, which are beyond the scope of this paper), women’s preferences for or experiences of place of birth did not vary across urban and rural settings. Some urban women did express that they had a choice in health-care facilities and that they had compared delivery services between different hospitals, whereas the rural women did not suggest that they had choices or that they compared facilities. The city of Port-au-Prince has more than twice as many health facilities, more than three times as many nurses, and over seven times as many midwives as the entire southern department [33]. Nonetheless, we found the aversion to isolation in hospitals to be common across urban and rural groups and to be reported for multiple facilities in Port-au-Prince.

The 2016–17 Haitian Demographic Health Survey found transportation cost and distance to facilities to be the major obstacles for women to deliver in health facilities, i.e., the first and second delays [3]; however, this survey did not report directly on the hospital-birth experience. Our study found compassionate maternity care to be an important factor in the decision about where to give birth. We found that the experience of isolation negatively affected women’s perceptions of the hospital birth; it also eroded confidence in choosing a hospital for the location of future births. Negative perceptions of facility-based maternity care can also have a psychological impact that affects future reproductive health decisions, such as seeking antenatal or postnatal care [34]. Distinct from women’s feelings about isolation is also the implication that isolation indicates a monitoring delay in the timely detection of complications and in appropriate interventions to prevent or treat a life-threatening complication, such as preeclampsia. Certainly, the issue of isolation is not attributable to provider attitude or behavior alone. As has been noted in several studies, Haitian facilities are understaffed, overcrowded, and lack basic supplies that cause further delays in the administration of appropriate care [35, 36]. We argue, however, that it would be possible to address these issues (without additional significant financial resources) if the clinical training curriculum were to include respectful and compassionate care as an integral part of the current standard of maternal care in Haitian facilities.

Women expressed a preference for home births over facility births, based on the physical support and care they receive during labor and delivery. They noted a lack of care, leading to emotionally and physically isolating experiences in facilities*.* Having continuous social support during birth is also recognized as an important intervention by the WHO [37]. Research shows that women with continuous support are less likely to use any intrapartum analgesia, are more likely to have a spontaneous vaginal birth, and are less likely to report negative ratings of or feelings about their childbirth experience. They also tend to have shorter labors and are less likely to have a cesarean or a baby with a low Apgar score [38]. Prata and colleagues recommend training traditional birth attendants or community health workers to deliver life-saving interventions during home births, but this approach is currently not supported by Haiti’s public health ministry (Ministère de la Santé Publique et de la Population, or MSPP) [39]. MSPP’s program for maternal and newborn health in Haiti exclusively advocates for facility births with skilled birth attendants [40]. Therefore, within the context of this policy and our data from this study, it would be more feasible to identify and integrate interventions that would encourage facility births by improving the overall experience of the facility birth.

One realistic recommendation for immediately improving the facility birth experience would be to allow the women to have a labor companion. Labor companions could help provide information and bridge communication with providers; advocate on behalf of the women; and provide physical and emotional support [41]. While our research did not focus specifically on solutions, we find women’s isolating facility birth experiences to suggest a need for a labor companion who can provide similar support to what they most value in home births; however, at the same time, we are aware that such a person should be trained, prepared, and have an approved role in a clinical setting. In high-volume maternity wards, for instance, a care navigator would be helpful during the labor and delivery, as well as in the postpartum period, to provide clinical observation, communicate with family, and help the patient through the labor and delivery process [42, 43]. The WHO’s recommendations on Intrapartum Care for a Positive Childbirth include choice of birth position and mobility for low-risk births in the facility setting—a recommendation that is highly applicable for Haitian facilities, given that women in both rural and urban areas noted the inability to move and having to lie down for delivery as important constraints in facility births [44]. Another approach would be to focus on staff training, particularly on staff empowerment, to address structural restrictions that impede better patient-provider interactions. For example, a comparison of two hospitals in Tanzania showed that, in similarly constrained circumstances, providers’ and women’s experiences were much more positive when nurse-midwives and nurse managers felt they were effective in influencing their environment and outcomes in the hospital [45]. Other policy-driven approaches may be enacted for a more systematic impact, such as the specific inclusion of respectful maternity care in the quality-of-care standards for maternal health [46]. In Brazil, for example, a national law allows all women in the public and private sectors to have a labor companion of their choice during all antenatal, labor, delivery, and postpartum care visits to a facility [47].

### Strengths and limitations

We note that national survey data have documented financial barriers to facility delivery in Haiti, including cost of medical care, facility fees, and cost of transportation [3]. In other settings, the expectation of disrespect from physicians and hospital staff has been shown to impede hospital births [15–17, 41]. Yet, few studies have analyzed women’s perspectives on the facility birth experience in Haiti and, to our knowledge, none have identified the role that isolation plays in that experience versus a home birth in both urban and rural settings. Given that this is a qualitative study with a limited sample size, and that there is limited standardization of medical care in Haiti, the experiences of isolation (detailed above) may not necessarily be transferable to all facility settings, such as those that are private, well-funded, or uncongested. The women we spoke with were poor and relied on public sector facilities; however, one cannot assume that the private sector necessarily has different outcomes. For example, one study from India found that the private sector was even stricter about allowing companions during delivery [16]. Future studies that focus on Haitian women with higher incomes or with access to a range of public and private hospital facilities would supplement our findings. The significance that a supportive environment plays in a woman’s assessment of her birthing experience(s) reinforces current modifications to global health policy. Our findings reinforce the goal, adopted by the WHO and others, to provide women in labor with a “humane, supportive” environment [13, 48]. We hope that our study will motivate Haitian practitioners to address the experience of isolation as contradictory to a humane and supportive environment. We expect that additional research into this area will further define factors that contribute to feelings of isolation and will further evaluate evidence-based procedures to address isolation, while also acknowledging and accounting for the pressures placed on facilities in terms of space, crowding, and costs.

We also note a few further limitations of our study. We did not distinguish between the types of facilities women visited for deliveries. While women always used the word “hospital,” some of these were health centers, which would have varying staff capacity and offer different obstetric services than hospitals [49]. We could not say from the focus groups whether the comments were primarily about health centers or hospitals and, therefore, refer to the place of delivery using “facility” as a unified term. We also are aware that, in a group setting, women may have preferred to express a preference for a facility birth since facility births tend to indicate higher socioeconomic status in Haiti. In addition, although it is culturally appropriate to begin conversations by asking how one is doing and what one is doing, this may have elicited information about mood, source of income, and family life that biased the respondents toward demonstrating a certain socioeconomic status. However, we believe that this introduction was essential, because it served to create camaraderie among the group and to aid women in their ability to connect with one another; for instance, as they were able to connect over their shared experiences as mothers without formal employment. We also understand that women’s preferences for facility births and their complaints about facility births may have been shaped by their relationships with the public health clinic and our role as foreign researchers tied to health projects. In other words, the women could ascertain that we were both interested in both encouraging facility births and improving the facility birth experience, which is why we also chose to include women who gave birth at home and those who were currently pregnant. Notably, while most women expressed a preference for a facility birth when asked directly, it was interesting that they tended to modify their preference over the course of discussing their facility-birth experiences with others. As noted, first-time pregnant women were included to understand where they were planning to deliver and why. While this added an interesting perspective to the discussion, we realize that their understanding was not based on their own experiences and that the data they provided reflected impressions they gained from other women in their families or communities; however, their impressions did not contradict the experience of others.

Another limitation could have been the group format, which may have amplified the discussion towards women’s shared experiences. Despite this, we believe the focus group format was the best approach, as it allowed us to establish shared experiences for a diverse group of women in a relatively short timeframe and helped us to identify overarching themes that could form the basis for targeted research in the future. In cases of emotionally sensitive outcomes (such as a severe complication, stillbirth, or a neonatal death), an in-depth interview format would be more appropriate. We also note that we may have some bias in the sample since participants were selected by the clinic staff, and they could have chosen women who were more likely to come for antenatal care, were closer in proximity to the clinic, or who had not experienced a severe complication or death in their most recent pregnancy. This could have biased the sample toward healthier women; however, we do not think it biased the sample to share different stories than what they had experienced. We did observe that the urban sample tended to be younger and quieter, needing more promoting than the rural sample.

We did not provide participants with an opportunity to review transcripts and provide feedback, in part due to concerns over low rates of literacy in the rural setting and in part due to difficulties in locating participants again, especially in the urban setting. We would have valued the chance to work more closely with the participants. We did provide the transcripts to the local clinical staff, with whom we worked closely. We plan to continue our collaboration toward determining how our findings can inform practice and policy, such as presenting the findings to the MSPP.

## Conclusions

Our overall objective for this study was to elucidate the factors affecting a woman’s decision to give birth in a health facility or at home. While many women expressed the desire to deliver at a hospital in the case of a pregnancy complication, when we asked what it was like to give birth in a facility, they reported largely negative experiences. The existing literature recognizes respectful care as an important component of right to care, supportive care, and trust. What has been under-appreciated is the support structure, or lack thereof, that women encounter in hospitals. This factor—what we are calling isolation—stands at the nexus of respect and care, of promoting positive social relations and quality care. Our research, thus, highlights the importance of companionship, support, and attention during labor and delivery as an intersectional component of respectful maternity care; however, we also recognize the importance of structural barriers that prevent health providers, especially nurses and midwives, from delivering quality, respectful care. Improvements need to be made in the interpersonal communication and care provision by obstetric providers that is conducive with the expectations and needs of pregnant women. We recommend that current maternal health promotion programs in Haiti consider how to establish better models of respectful and compassionate maternity care into any interactions a pregnant woman has within the health-care system, and to share their findings while advocating for respectful care for all Haitian women.

## Supplementary information

**Additional file 1:** COREQ checklist.

**Additional file 2:** Question guide for focus groups.

**Additional file 3:** Information sheet/consent form.

## Data Availability

The datasets used and analyzed for this study are available from the corresponding author upon reasonable request.

## Abbreviations

COREQ: Consolidated criteria for documenting qualitative research
FGD: Focus group discussion
GHESKIO: Haitian Study Group for Kaposi’s Sarcoma and Opportunistic Infections
MSPP: Ministère de la Santé Publique et de la Population
MWH: Maternity waiting homes
SUCO: Solidarity, Union, and Cooperation
WHO: World Health Organization

## References

[1] Kassebaum NJ, Bertozzi-Villa A, Coggeshall MS, Shackelford KA, Steiner C, Heuton KR (2014). Global, regional, and national levels and causes of maternal mortality during 1990–2013: a systematic analysis for the global Burden of disease study 2013. Lancet.

[2] Lawn J, Shibuya K, Stein C (2005). No cry at birth: global estimates of intrapartum stillbirths and intrapartum-related neonatal deaths. Bull World Health Organ.

[3] Institut Haïtien de l’Enfance (IHE), ICF. Enquête Mortalité, Morbidité et Utilisation des Services (EMMUS -VI 2016-2017). Pétion -Ville, Haïti, et Rockville, Maryland, USA: IHE et ICF; 2018.

[4] World Health Organization. Defining competent maternal and newborn health professionals. Geneva: WHO; 2018.

[5] MSPP. Determinants du choix du lieu de l’accouchement par les femmes Haitiennes/determinants of choice of place of delivery among Haitian women. Port au prince, Haiti: MSPP; 2015.

[6] Thaddeus S, Maine D (1994). Too far to walk: maternal mortality in context. Soc Sci Med 1982.

[7] van Lonkhuijzen L, Stekelenburg J, van Roosmalen J. Maternity waiting facilities for improving maternal and neonatal outcome in low-resource countries. In: The Cochrane Collaboration, editor. Cochrane Database of Systematic Reviews [Internet]. Chichester, UK: John Wiley & Sons, Ltd; 2009. Available from: http://doi.wiley.com/10.1002/14651858.CD006759.pub2 [cited 2019 3 Jan] 10.1002/14651858.CD006759.pub2 19588403

[8] Kaiser J, Fong R, Ngoma T, Lee D, Sakanga V, Bwalya M, et al Influence of maternity waiting homes on health workers’ perceived ability to deliver care in rural Zambia: a qualitative analysis. In: Developing the Global Health Workforce. San Diego, CA; 2018.

[9] Haitian Health Foundation. Center of Hope Maternity Waiting Home [Internet]. Haitian Health Foundation. 2015; Available from: https://www.haitianhealthfoundation.org/center-of-hope/ [cited 2019 5 Sep].

[10] Partners in Health. New Maternal Waiting Home in Haiti Offers “Priceless” Care [Internet]. Partners In Health. 2018; Available from: https://www.pih.org/article/new-maternal-waiting-home-haiti-offers-priceless-care [cited 2019 Sep 4].

[11] Montagu D, Sudhinaraset M, Diamond-Smith N, Campbell O, Gabrysch S, Freedman L (2017). Where women go to deliver: understanding the changing landscape of childbirth in Africa and Asia. Health Policy Plan.

[12] World Health Organization, Ariadne Labs. WHO safe childbirth checklist implementation guide: improving the quality of facility-based delivery for mothers and newborns. Geneva. 2015.

[13] The White Ribbon Alliance. Respectful Maternity Care: The Universal Rights of the Childbearing Women [Internet]. Washington DC; 2011. Available from: https://www.who.int/woman_child_accountability/ierg/reports/2012_01S_Respectful_Maternity_Care_Charter_The_Universal_Rights_of_Childbearing_Women.pdf [cited 2019 6 Mar]

[14] Bohren MA, Vogel JP, Hunter EC, Lutsiv O, Makh SK, Souza JP (2015). The mistreatment of women during childbirth in health facilities globally: a mixed-methods systematic review. PLoS Med.

[15] Oluoch-Aridi J, Smith-Oka V, Milan E, Dowd R. Exploring mistreatment of women during childbirth in a peri-urban setting in Kenya: experiences and perceptions of women and healthcare providers. Reprod Health [Internet]. 2018;15 Available from: https://www.ncbi.nlm.nih.gov/pmc/articles/PMC6296108/ [cited 2019 5 Sep]. 10.1186/s12978-018-0643-z PMC6296108 30558618

[16] Sharma G, Penn-Kekana L, Halder K, Filippi V (2019). An investigation into mistreatment of women during labour and childbirth in maternity care facilities in Uttar Pradesh, India: a mixed methods study. Reprod Health.

[17] Orpin J, Puthussery S, Davidson R, Burden B (2018). Women’s experiences of disrespect and abuse in maternity care facilities in Benue state. Nigeria BMC Pregnancy Childbirth.

[18] Theuring S, Koroma AP, Harms G (2018). “In the hospital, there will be nobody to pamper me”: a qualitative assessment on barriers to facility-based delivery in post-Ebola Sierra Leone. Reprod Health.

[19] Consolidated criteria for reporting qualitative research (COREQ): a 32-item checklist for interviews and focus groups | International Journal for Quality in Health Care | Oxford Academic [Internet]. Available from: https://academic.oup.com/intqhc/article/19/6/349/1791966 [cited 2019 14 May] 10.1093/intqhc/mzm042 17872937

[20] Focus Group. In: Encyclopedia of Survey Research Methods [Internet]. 2455 Teller Road, Thousand Oaks California 91320 United States of America: Sage Publications, Inc.; 2008. Available from: http://methods.sagepub.com/reference/encyclopedia-of-survey-research-methods/n192.xml [cited 2019 3 Sep]

[21] Institut Haïtien de l’Enfance (IHE). Socio-demographic profile of the population served by the Saint Anne Clinic. Port au Prince, Haiti: Institut Haïtien de l’Enfance; 2017.

[22] GHESKIO. Zika in pregnant women and infants [unpublished]. Port au prince, Haiti: GHESKIO; 2018.

[23] Urrutia RP, Merisier D, Small M, Urrutia E, Tinfo N, Walmer DK (2012). Unmet health needs identified by Haitian women as priorities for attention: a qualitative study. Reprod Health Matters.

[24] White K, Small M, Frederic R, Joseph G, Bateau R, Kershaw T (2006). Health seeking behavior among pregnant women in rural Haiti. Health Care Women Int.

[25] Babalola SO (2014). Factors associated with use of maternal health services in Haiti: a multilevel analysis. Rev Panam Salud Publica Pan Am J Public Health.

[26] Strauss A, Corbin J. Grounded theory in practice. Thousand Oaks, CA: Sage Publications; 1997.

[27] Provalis Research. QDA Miner Lite. Provalis Research; 2018. Available from: https://provalisresearch.com/products/qualitative-data-analysis-software/freeware/

[28] Shiferaw S, Spigt M, Godefrooij M, Melkamu Y, Tekie M (2013). Why do women prefer home births in Ethiopia?. BMC Pregnancy Childbirth.

[29] Fotso J-C, Ezeh AC, Essendi H (2009). Maternal health in resource-poor urban settings: how does women’s autonomy influence the utilization of obstetric care services?. Reprod Health.

[30] Ahmed S, Creanga AA, Gillespie DG, Tsui AO (2010). Economic status, education and empowerment: implications for maternal health service utilization in developing countries. PLoS One.

[31] Anderson FWJ, Morton SU, Naik S, Gebrian B (2007). Maternal mortality and the consequences on infant and child survival in rural Haiti. Matern Child Health J.

[32] Woldemicael G, Tenkorang EY (2010). Women’s autonomy and maternal health-seeking behavior in Ethiopia. Matern Child Health J.

[33] Institut Haïtien de l’Enfance (IHE). HAÏTI Évaluation des Prestations des Services de soins de Santé (EPSS) 2017–2018. Rapport des Indicateurs Clés. Rockville, MD: The DHS Program, ICF; 2018.

[34] Mirkovic KR, Lathrop E, Hulland EN, Jean-Louis R, Lauture D, D’Alexis GD (2017). Quality and uptake of antenatal and postnatal care in Haiti. BMC Pregnancy Childbirth..

[35] Winter R, Yourkavitch J, Wang W, Mallick L. Assessment of health facility capacity to provide newborn care in Bangladesh, Haiti, Malawi, Senegal, and Tanzania. J Glob Health [Internet]. 7(2) Available from: https://www.ncbi.nlm.nih.gov/pmc/articles/PMC5804038/ [cited 2019 25 Apr]. 10.7189/jogh.07.020509 PMC5804038 29423186

[36] Wang W, Winner M, Burgert-Brucker CR (2017). Limited service availability, readiness, and use of facility-based delivery Care in Haiti: a study linking health facility data and population data. Glob Health Sci Pract.

[37] Shakibazadeh E, Namadian M, Bohren M, Vogel J, Rashidian A, Nogueira Pileggi V (2018). Respectful care during childbirth in health facilities globally: a qualitative evidence synthesis. BJOG Int J Obstet Gynaecol.

[38] Bohren MA, Hofmeyr GJ, Sakala C, Fukuzawa RK, Cuthbert A. Continuous support for women during childbirth. Cochrane Database Syst Rev [Internet]. 2017;(7) Available from: https://www.cochranelibrary.com/cdsr/doi/10.1002/14651858.CD003766.pub6/full [cited 2019 31 Mar]. 10.1002/14651858.CD003766.pub6 PMC6483123 28681500

[39] Prata N, Passano P, Rowen T, Bell S, Walsh J, Potts M (2011). Where there are (few) skilled birth attendants. J Health Popul Nutr.

[40] MSPP. Plan Stratégique National de Santé Sexuelle et Reproductive 2018-2022/Strategic Plan for Sexual and Reproductive Health (2018–2022). Port au Prince, Haiti: MSPP; 2018.

[41] Bohren MA, Berger BO, Munthe-Kaas H, Tunçalp Ö. Perceptions and experiences of labour companionship: a qualitative evidence synthesis. Cochrane Database Syst Rev [Internet]. 2019; 2019(3). Available from: https://www.ncbi.nlm.nih.gov/pmc/articles/PMC6422112/ [cited 2019 5 Sep] 10.1002/14651858.CD012449.pub2 PMC6422112 30883666

[42] Austad K, Chary A, Martinez B, Juarez M, Martin YJ, Ixen EC, et al. Obstetric care navigation: a new approach to promote respectful maternity care and overcome barriers to safe motherhood. Reprod Health [Internet]. 2017;14 Available from: https://www.ncbi.nlm.nih.gov/pmc/articles/PMC5683321/ [cited 2019 Apr 20]. 10.1186/s12978-017-0410-6 PMC5683321 29132431

[43] Yee LM, Martinez NG, Nguyen AT, Hajjar N, Chen MJ, Simon MA (2017). Using a patient navigator to improve postpartum Care in an Urban Women’s health clinic. Obstet Gynecol.

[44] WHO. WHO recommendations- Intrapartum care for a positive childbirth experience [Internet]. Geneva: World Health Organization; 2019. Available from: https://apps.who.int/iris/bitstream/handle/10665/272447/WHO-RHR-18.12-eng.pdf?ua=1 30070803

[45] Tibandebage P, Kida T, Mackintosh M, Ikingura J (2016). Can managers empower nurse-midwives to improve maternal health care? A comparison of two resource-poor hospitals in Tanzania. Int J Health Plann Manag.

[46] Manu A, Arifeen S, Williams J, Mwasanya E, Zaka N, Plowman BA (2018). Assessment of facility readiness for implementing the WHO/UNICEF standards for improving quality of maternal and newborn care in health facilities - experiences from UNICEF’s implementation in three countries of South Asia and sub-Saharan Africa. BMC Health Serv Res.

[47] Diniz CSG, d’Orsi E, Domingues RMSM, Torres JA, Dias MAB, Schneck CA (2014). Implementação da presença de acompanhantes durante a internação para o parto: dados da pesquisa nacional Nascer no Brasil. Cad Saúde Pública.

[48] World Health Organization. WHO recommendation on respectful maternity care during labour and childbirth [Internet]. Geneva: WHO; 2018. Available from: https://extranet.who.int/rhl/topics/preconception-pregnancy-childbirth-and-postpartum-care/care-during-childbirth/who-recommendation-respectful-maternity-care-during-labour-and-childbirth [cited 2019 14 May]

[49] Ministère, de la Santé Publique et de la Population (MSPP), MSPP. Le Pacquet Essentiel de Services [Internet]. Port au Prince, Haiti: MSPP; 2015. Available from: https://mspp.gouv.ht/site/downloads/Manuel%20du%20PES%20Lancement%201er%20Septembre%202016%20compressed.pdf [cited 2018 23 Sep]

