# Supplementary material for: Perceptions of isolation during facility births in Haiti - a qualitative study
Source: Reprod Health. 2019 Dec 27;16:185. doi: 10.1186/s12978-019-0843-1 (PMC6935234; doi:10.1186/s12978-019-0843-1)
Supplement: Supplementary file 2 — Additional file 2: Question guide for focus groups. [file 12978_2019_843_MOESM2_ESM.docx]

**Supplemental File 2-Focus Group Question Guide**

**REQUEST FOR CONSENT:** Introduce study question and procedures. Obtain oral consent.

(Start recording)

**GROUND RULES:**

Before we start, I would like to remind you that there are no right or wrong answers in this discussion. We are interested in knowing what each of you think, so please feel free to be frank and to share your point of view, regardless of whether you agree or disagree with what you hear. It is very important that we hear all your opinions.

You probably prefer that your comments not be repeated to people outside of this group. Please treat others in the group as you want to be treated by not telling anyone about what you hear in this discussion today.

Let's start by going around the circle and having each person introduce herself by telling us how you are doing, how you family is doing, and what your activities are. Members of the research team also introduced themselves and describe each of their roles. Can you tell us about your recent birth experience including what number of birth this is for you and the place of delivery? If pregnant, can you tell us about your pregnancy, including what number of pregnancy this is for you and where you plan to deliver?

**Research Question:** Where do you prefer to give birth, and why?

- Proximity: Does the location of your house relative to the facility or the midwife affect your decision about where to give birth?
- Transportation: How do you get to the hospital, or how does the midwife come to you? Does the factor of transportation affect your decision about where to give birth?
- History of Care: Do you have any experiences with prenatal care at the hospital or with a midwife? How did these previous experiences affect your decision for where to give birth?
- Financial Concerns: How does the factor of cost influence your decision about where to give birth?
- Societal Values and Cultural Norms: What do you think most people believe is the proper place to give birth for Haitian women? Does this societal expectation influence your preference about where to give birth?
- Insecurity: Where do you think it is safe for you to give birth? Does the threat of violence or insecurity influence your decision?
- People involved: In your experience, who contributes to the decision about where you give birth? (Probe: Midwives? Husbands? Mothers? Fathers? Other family members? Religious or community leaders? Others?)

**Birth** **Experience/Perceptions:** Speaking from experience or your knowledge of the experience, how would you describe an institutional delivery? A home birth?

- What is it like? What happens? Walk us through the process?

**Probe of Contradiction:** Many people have expressed that they desire to give birth in a hospital, but at the same time, told us that they had negative experiences in the hospital. Can you explain this more?

- What do peole like about the hospital? What do you not like?
- What do you like about the home birth that is not present in the hospital?
- What can go well in the hospital? And not well?
- Would you have a hospital birth again?

**Policy Suggestions:** What could be done to improve the hospital birth experience for women?

- What would you change about the hospital birth experience? How would you change it?
- What aspects of the home birth could be brought into the hospital?
- Do you think these changes are feasible? What challenges might the hospital face?

**CONCLUSION:**

We realize that the topics discussed in this focus group may not account for the unique experiences of each member of the group or may have raised sensitive issues for you. If you would like to talk further about your individual birth stories, we are available to do so after a break for refreshments.

**MANDE KONSANTMAN:** Prezante kesyon rechèch ak pwosesis, epi mande konsantman.

(Komanse enrejistreman)

**Regleman yo:**

Avan nou komanse, m ta renmen di ke pa gen repons korrekt oswa pa korekt. Nou enterese nan tout lide nou, ki donk ou lib pou pataje tout sa ou panse, menm si lòt moun ka pa dakò. Li enpòtan pou nou konnen opinion tout moun yo.

N ap tout mete tèt ansanm pou respekte youn ak lòt. Nou ta renmen kenbe diskisyon sa a prive. Se posib w ap di yon bagay ou pa vle lòt moun tande ki pa nan gwoup la. Epi s aka vre pou lòt dam yo tou. Tanrpi pa repete sa ou tande deyo gwoup la.

N ap komanse ak chak moun prezante tèt ou. Ou pa dwe di nou non ou, men eske ou ka di nou koman ou ye, koman fanmi ou a ye, epi ki aktivite ou genyen? Chèchè pral prezante tèt yo tou. Eske ou ka di nou koman denyè akouchman te pase, oswa koman gwoses la ap mache? Kombyen petit sa ye pou ou? Ki kote ou te/vle akouche? Koman sa te ye oswa koman ou prevwa sa—nan yon mo?

**Research Question:** Ki kote ou prefere akouche? Poukisa?

- Distans: Eske kote kay ou ye pa rapò ak kote opital ye oswa matwon ye enfliyanse kibò ou vle akouche?
- Transpò: Koman ou ka rive nan opital, oswa koman matwon ka vin jwenn ou, epi eske sa afekte kibò ou prefere akouche?
- Istwa Swen Medikal: Eske opital la oswa matwon nan te bay ou swen maternèl oswa prenatal? Koman eksperyans sa yo te antre nan desisyon ou fè pou lokal akouchamn?
- Kesyon Moyen: Koman kesyon moyen enfliyanse kibò ou prefere akouche?
- Opinyon Piblik: Kibò ou panse piblik la kwe yon fanm dwe akouche nan sosyete Ayisyen? Eske opinyon piblik enfliyanse kibò ou vle akouche?
- Ensekirite: Kibò w ap jwenn plis sekerite nan akouchman? Eske kesyon ensekirite oswa vyolans antre nan desisyon w ap fè pou lokal akouchman?
- Rezo Sosyal: Nan eksperyans pa ou a, kiyès k ap ede ou deside kibò pou akouche? (Matwon? Mari? Manman? Papa? Lòt fanmi? Lidè relijye oswa kominotè? Lòt moun?)

**Eksperyans/Pèsepsyon Akouchman:** Dapre eksperyans ou oswa dapre lide ou genyen sou akouchman, koman w ap dekri yon akouchamn nan opital? Akouchman lakay?

- Koman sa ye? Ki sa ka fèt?

**Fokis sou Kontradiksyon:** Anpil moun te di yo vle akouche opital, men lè nou rakonte koman sa ye nan opital, nou te pataje anpil move eksperyans?

- Ki sa nou renmen nan akouchman opital? Ki sa nou pa renmen?
- Ki sa ou renmen nan akouchman lakay ki pa nan akouchman opital?
- Ki sa ka byen pase nan opital? Ki sa pa byen pase?
- Eske ou t ap akouche opital ankò?

**Sijesyon sosyal:** Ki sa nou ka fè pou amelyore akouchman opital?

- Ki sa ou t ap chanje nan akouchman opital? Koman w ap fè chanjamn sa yo?
- Ki aspe akouchman lakay ou panse dwe antre nan akouchman opital?
- Eske ou panse chanjman sa yo ka fèt? Ki obstak nou ka genyen?

**Konkklisyon:**

Nou komprann ke sije nou te diskite yo pa ka kapte tout detay nan chak akouchman, epi nou komprann ke sije sa a delika ak difisil pou nou diskite. Si ou vle pale plis ak nou, n ap disponib apre yon ti poz pou bwe ak manje yon bagay.
