# Supplementary material for: Perceptions of isolation during facility births in Haiti - a qualitative study
Source: Reprod Health. 2019 Dec 27;16:185. doi: 10.1186/s12978-019-0843-1 (PMC6935234; doi:10.1186/s12978-019-0843-1)
Supplement: Supplementary file 3 — Additional file 3: Information sheet/consent form. [file 12978_2019_843_MOESM3_ESM.doc]

DARTMOUTH COLLEGE

***Experiences of Pregnancy, Labor, and Delivery in the Rural South of Haiti***

RESEARCH PROJECT INFORMATION SHEET/CONSENT FORM

This research project is being conducted by Alka Dev from Dartmouth-Hitchcock Medical Center and Chelsey Kivland from the Anthropology Department at Dartmouth College. We also have the assistance of representatives from your community. We plan to speak with pregnant and postpartum women to learn how their decision-making process around place of birth. We want to learn about women’s pregnancy and birth experiences at home and in the hospital. We aim to better understand why women decide to deliver at a facility, or not. Our goals is to improve pregnancy and prenatal care in the region.

Your participation is voluntary. Participation involves taking part in a focus group and potential follow-up discussions. You may choose not to participate in any or all aspects of the research, as well as not answer any or all questions you are asked during the research. With your permission, your consent, the focus group will be audio recorded. You may request that the recording be stopped at any time. The audiotapes will contain none of your identifying information. The audiotapes will be transcribed and held at Dartmouth College for the duration of research collection and after for archival purposes.

The information collected will be maintained confidentially. Names and other identifying information will not be used in any presentation or paper written about this project, unless you explicitly request otherwise.

Do you have any questions? If you do have a question at a later date, please feel free to contact us.

Alka Dev

330W Borwell

1 Medical Center Drive

Lebanon, NH 03756

Alka.dev@hitchcock.org

Chelsey Kivland

6047 Silsby Hall

Hanover, NH 03768

011 603-646-3334

ckivland@gmail.com

Now, I will turn on the audio recorder and ask if you agree to participate in the study. This recording will serve as your consent to participate. Again, you can choose to not participate. If you would like to participate, please respond by saying, “Yes.”

DARTMOUTH COLLEGE

***Gwosès ak Akouchman nan Zòn Sud Peyi Dayiti***

ENFOMASYON SOU PWOJE RECHECH LA/FOM KONSANTMAN

Gen de moun ki pral dirije pwoje rechèch sa: Alka Dev ki se yon chèchè nan Dartmouth-Hitchcock Medical Center ak Chelsey Kivland ki se yon pwofesè nan Depatman Antwopoloji nan Dartmouth College. Epi tou nou kontan paske mamb kominote nou a k ap asiste mou nan rechech sa. Nou ta renmen pale ak fi ki te gen tan fè petit ak moun ki gen pou yo fè petit. N ap mande ki eksperyans famn yo genyen pandan akouchman. Nou enterese aprann koman sa ye ni pou fanm ki akouche lakay yo ni sila yo ki akouche lopital. Nou fè rechèch sa a paske nou vle pi byen komprann poukisa fanm yo deside akouche nan optital oswa pa akouche nan opital. Vizyon nou se pou nou ameliyore swen avan ak pandan akouchman pou tout fanm ki abite isi.

Patisipasyon ou nan rechèch sa se yon bagay volontè. Gen plizyè faz nan pwoje a: gwoup fokis (w ap diskite sije ak lòt patisipan) epi, si li nesesè, n ap fè seivi ak plis diskisyon. Si ou pa vle patispe, ou ka di non. Ou ka di ou pa dakò ak yon pati rechèch la oswa yon kesyon nou poze pandan rechèch la. Si ou ba nou pèmisyon ou—sa vle di ou dakò—n ap enrejistre enfòmasyon ke nou konpile nan fokis gwoup la. Nenpòt lè ou ka mande pou nou fèmen aparey la si ou pa vle nou enrejistre yon bagay. Enrejistreman an ak tout nòt nou yo n ap mete yo nan yon kote sekeryize nan Dartmouth College.

Nou pral kenbe enfomasyon sa konfidantsyèl—sa vle di an sekrè. Nou p ap sèvi ak non ou oswa lòt detay pèsonèl ou nan rapò a, presantsayon, oswa lòt bagay nou ekri sou rechèch sa a—sòf si ou mande nou fè sa espesyalman. Eske ou gen kesyon? Si ou gen yon kesyon aprè nou fini nèt a rechech la, tanpri rele oswa ekri nou.

Alka Dev

330W Borwell

1 Medical Center Drive

Lebanon, NH 03756

Alka.dev@hitchcock.org

Chelsey Kivland

6047 Silsby Hall

Hanover, NH 03768

603-646-3334

ckivland@gmail.com

Kounye a, n ap louvri aparey anrejistreman nou epi n ap mande w si ou ta remen patisipe nan rechèch sa. Avan nou kòmanse anrejistreman an di nou si ou dakò oswa si ou pa dakò. Ankò, m ap respekte dwa ke ou genyen pou deside si ou pa vle patisipe. Men si ou dakò, tanpri di wi. Mèsi!
